# Biological influence of brain-derived neurotrophic factor (BDNF) on colon cancer cells

**DOI:** 10.3892/etm.2013.1330

**Published:** 2013-10-07

**Authors:** XIAOMEI YANG, TRACEY A. MARTIN, WEN GUO JIANG

**Affiliations:** 1 Metastasis and Angiogenesis Research Group, Cardiff University School of Medicine, Cardiff CF14 4XN, UK; 2 Cardiff University-Capital Medical University Joint Centre for Biomedical Research, School of Basic Medical Science, Capital Medical University, Beijing 100069, P.R. China; 3 Department of Biochemistry and Molecular Biology, Capital Medical University, Beijing 100069, P.R. China

**Keywords:** BDNF, colon cancer, proliferation, apoptosis

## Abstract

Brain-derived neurotrophic factor (BDNF) has been observed to be elevated in solid tumors including colorectal cancer. The present study aimed to investigate the effect of modulation of BDNF at the transcription level on the cellular function of colorectal cells and to increase our understanding of its biological role in human colon cancer. An investigation of a cohort of human colorectal tissues (tumor n=66; normal n=88) using quantitative PCR and immunohistochemistry demonstrated that BDNF is aberrantly expressed in human colon cancer and a significantly raised level of BDNF is associated with its stage at diagnosis. The expression profile of BDNF in human colon cancer cell lines was evaluated using RT-PCR. A set of anti-BDNF ribozymes were used to transfect colon cancer cells in order to generate BDNF knockdown cells to evaluate the effect on growth and apoptosis. BDNF gene transcripts were successfully detected in the colon cancer cell lines, Caco-2 and HRT18. BDNF knockdown in Caco-2 and HRT18 cell lines resulted in decreased rates of growth and proliferation. Analysis of apoptosis showed that cell apoptosis was increased. It is concluded that BDNF, a neurotrophic growth factor aberrantly expressed in cancers such as colon cancer, has a profound impact on the cellular behavior of colon cancer cells and that BDNF is associated with a reduction in the apoptosis of colon cancer. BDNF is therefore a potential therapeutic target in colon cancer and its effect in human colon cancer requires further investigation.

## Introduction

Colon cancer is one of the most common gastrointestinal sarcomas. The incidence of colon cancer in North America and Western Europe is higher than that in developing countries, but there is also a trend to increasing incidences in developing countries (1,2). The etiology of colon cancer remains unclear, but is believed to be closely associated with environmental factors, genetic factors and pre-cancerous diseases. The survival rate of patients with colon cancer is closely associated with the stage of the disease, early diagnosis and treatment, which would also contribute to improvement in survival and prognostic evaluation. The treatment of colon cancer includes surgery, chemotherapy, radiotherapy, immunotherapy and biotherapy. Due to its molecular and cellular diversification, the individual treatment of colon cancer has attracted much attention. In addition, >25% of colon cancer cases occur in patients with a family history of colon cancer. Prevention, monitoring or preventive treatment of individuals at high risk has become extremely important (3,4). The discovery of new colon cancer tumor markers and the mechanism underlying their modality may provide new targets for diagnosis and treatment.

The expression level and impact of brain-derived neurotrophic factor (BDNF) has been reported in several types of carcinomas, although how this protein exerts its effects remains to be identified (5). BDNF is a protein composed of 247 amino acids, which was initially separated and purified from pig brain (6). It is a member of the nerve growth factor family and is secreted by the target cells for BDNF, being important to the phenotypic behavior of neurons or neural system cancers. BDNF mediates cellular biological effects mainly through a cell surface tyrosine kinase receptor, tropo-myosin-related kinase B (TrkB). The extracellular N-terminal binds to BDNF, followed by receptor autophosphorylation to activate signaling pathways in cells (7).

In previous years it has been shown that BDNF expression is associated with different functions in non-neuronal solid tumors. BDNF expression controls cellular proliferation and survival, and is also connected to cell invasion via the secretion of matrix metalloproteinase (8–12). The PI3K/AKT and ERK pathways in tumor cells activated by BDNF may result in cells that are not sensitive to chemotherapeutic drugs (13,14).

As previously reported, screening of clinical samples has revealed that BDNF is highly expressed in colon carcinoma compared with non-tumor tissues (5). The present study aimed to investigate the biological roles of BDNF expression in human colon cancer. We investigated the expression level and the impact of BDNF in clinical samples. We observed high expression of BDNF in Caco-2, HRT18 and RKO human colon cancer cells and therefore anti-BDNF ribozymes were constructed to knock down the expression of BDNF in these cell lines. We then investigated the effects of BDNF knockdown on cellular behavior.

## Materials and methods

### Patient samples

Colorectal cancer tissues (n=66) and normal background tissues (n=88) were collected immediately after surgery and stored in a deep freeze until use. The presence of tumor cells in the collection tissues was verified by a consultant pathologist following hematoxylin and eosin (H&E) staining of the frozen sections. Details of the histology were obtained from pathology reports and together with basic patient demographics are shown in Tables I and II. The anonymous breast tissue samples were obtained within the guidelines of the appropriate ethics committee [University Hospital of Wales Trust (05/DMD/3562)]. informed patient consent was obtained following guidelines set out by the Human Tissue Act 2004, UK (http://www.legislation.gov.uk/ukpga/2004/30/contents).

### RNA preparation and reverse transcription PCR (RT-PCR)

Total cellular RNA was extracted from the cultured cells using Total RNA Isolation reagent (ABgene, Epsom, UK). The concentration of RNA was determined using an ultraviolet spectrophotometer (WPA UV 1101; Biotech Photometer, Cambridge, UK). cDNA was obtained by RT-PCR using a transcription kit (Sigma, Poole, UK). The quality of DNA was verified using GAPDH primers. The mRNA levels of BDNF were assessed using the BDNF primers. PCR were run on a GeneAmp PCR System 2400 thermocycler (Perkin-Elmer, Waltham, MA, USA). The PCR products were separated by 1% agarose gel, stained with ethidium bromide and images were captured with a digital camera mounted over a UV transluminator.

### Quantitative PCR (qPCR)

The mRNA level of BDNF gene expression was determined by the qPCR method using the prepared cDNA as the template and BDNF primers. An additional primer sequence was added to every qPCR system, known as the Z sequence (5′-ACT GAA CCT GAC CGT ACA-3′), which is complementary to the universal Z probe (Intergen Inc., Oxford, UK). The reaction was carried out in an IcyclerIQ™ (Bio-Rad, Hemel Hemstead, UK) using 96-well plates. GAPDH expression was used as an internal control. The reaction conditions were as follows: 94°C for 7 min, 80 cycles of 94°C for 15 sec, 55°C for 35 sec (the data capture step) and 72°C for 20 sec. The levels of the transcripts were generated from an internal standard that was simultaneously amplified with the samples.

### Immunohistochemistry of clinical samples

Cryostat sections of frozen tissues were cut at 6 μm, placed on Super Frost Plus slides (LSL UK, Rochdale, UK), air dried and fixed in a 50:50 solution of alcohol:acetone. The sections were then air dried again and stored at −20°C until used. Immediately prior to immunostaining, the sections were washed in buffered saline solution (BSS) for 5 min and treated with horse serum (Sigma-Aldrich Co. Ltd., Gillingham, UK) for 20 min as a blocking agent to non-specific binding. The sections were stained using BDNF antibody (Santa Cruz Biotechnology, Inc., Santa Cruz, CA, USA). Negative controls were used where necessary. The primary antibody was used at 1:100 dilution for 60 min and the sections were washed in buffer. The secondary biotinylated antibody at 1:100 dilution (Universal secondary, Vectastain Elite ABC; Vector Laboratories Inc., Burlingame, CA, USA) was added (in horse serum/buffer solution) for 30 min, followed by numerous washings. Avidin/biotin complex (Vector Laboratories Ltd., Peterborough, UK) was added for 30 min and followed by washes. Diaminobenzidine (Sigma-Aldrich Co. Ltd.) was used as a chromogen to visualize the antibody/antigen complex. Sections were counterstained with Mayer's hematoxylin for 1 min, dehydrated, cleared and mounted in DPX.

### Cell lines

The human colon cancer cell lines, Caco-2, HRT18 and RKO were obtained from the European Collection of Animal Cell Cultures (ECACC, Salisbury, England). Cells were maintained in Dulbecco's modified Eagle's medium (DMEM) containing 10% fetal calf serum, 100 U/ml penicillin and 100 μg/ml streptomycin (Gibco BRC, Paisley, UK) at 37°C and 5% CO_2_.

### Knockdown of BDNF expression using ribozyme and screening of stable transfected cell line

Ribozymes targeted to human BDNF transcription levels were designed based on the secondary structure of the gene generated using the Zuker RNA mFold program (University of Albany, New York, NY, USA). The ribozymes were accordingly synthesized and then cloned into pEF6/V5-his-Topo T/A vector (Invitrogen, Paisley, UK) and transfected into Caco-2 and HRT18 cells using an Easyjet Plus electroporator (EquiBio, Kent, UK). Following selection with culture medium containing 5 μg/ml blasticidin (Sigma-Aldrich Co. Ltd.), the verified transfectants were cultured in maintenance medium containing 0.5 μg/ml blasticidin. The primer sequences of the BDNF ribozymes were as follows: 5′-CTG CAG TTG GCC TTT TGA TAC AGG GAC CTT TTC AAG GAC TGT CTG ATG AGT CCG TGA GGA-3′ and 5′-ACT AGT GCA GTG GAC ATG TCG GGC GGG ACG GTT TCG TCC TCA CGG ACT-3′.

### Cell growth assay

Colon cancer cell growth rates were assessed using an *in vitro* growth assay. Cells were planted in sextuplicate into 96-well plates at a density of 2,000 cells per well. The plates were then incubated for 24, 48, 72 and 120 h before being fixed in 4% formaldehyde (v/v) and stained with 0.5% (w/v) crystal violet. The crystal violet stain was then extracted using 10% acetic acid (v/v) and cell density was determined by measuring the absorbance of this solution at 540 nm using a Bio-Tek ELx800 multi-plate reader (Bio-Tek Instruments Inc., Winooski, VT, USA).

### Flow cytometric analysis of apoptosis

All cells, including those floating in the culture medium, were harvested following incubation. Cells were washed in cold BSS and resuspended in 1X annexin V- binding buffer at a density of 1×10^6^ cells/ml after centrifugation for 8 min at 13,000 × g. Fluorescein isothiocyanate (FITC)-annexin V (5 μl) and 1 μl propidium iodide (PI) working solution (100 μg/ml; Molecular Probes, Eugene, OR, USA) were added to 100 μl cell suspension. After a 15-min incubation at room temperature, 400 μl 1X annexin V-binding buffer was added and mixed in gently, and the samples were immediately kept on ice. The stained cells were analyzed using a flow cytometer and FlowMax software (Partec UK Ltd., Canterbury, UK).

### Statistical analysis

Statistical analysis was performed using SPSS software (SPSS, Inc., Chicago, IL, USA). P<0.05 was considered to indicate a statistically significant result.

## Results

### Expression of BDNF in human colon cancer

**Figure 1:**
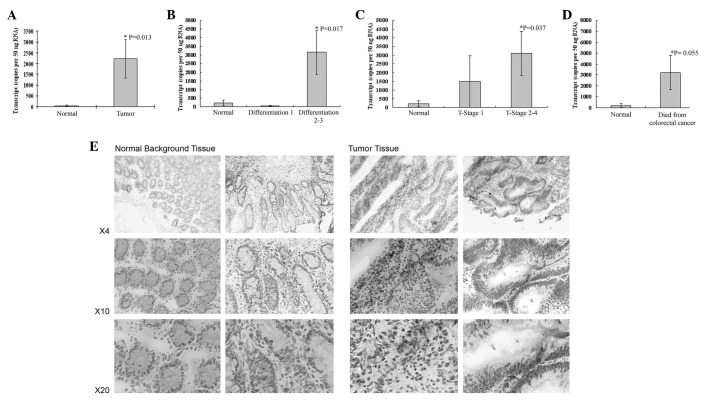
BDNF levels in human colorectal tumor tissues. Values represent the true copy number of mRNA transcripts. (A) Levels of BDNF transcript between normal and tumor tissues. ^*^Normal vs. tumor. (B) Levels of BDNF transcript in differentiated tumors. ^*^Normal vs. differentiation 2–3. (C) BDNF transcript levels and T-staging. ^*^Normal vs. T-Stage 2–4. (D) Levels of BDNF transcript and patient survival. ^*^Normal vs. succumbed to colorectal cancer. (E) Representative immunohistochemistry of BDNF protein in colorectal normal/background and tumor tissues. BDNF, brain-derived neurotrophic factor.

BDNF was observed to be expressed in colon cancer tissues and normal tissues. BDNF mRNA expression levels were detected using qPCR. Significantly higher mRNA levels were observed in colon cancer tissues compared with normal tissues (P=0.017; Fig. 1A). BDNF expression was significantly increased with increasing differentiation of colorectal tumors (well differentiated tumors versus moderate/poorly differentiated, P=0.017; Fig. 1B) and also increased with increasing T-staging (T-stage 1 versus 2 and 3, P=0.037; Fig. 1C). Patients who succumbed to colorectal cancer also had elevated levels of BDNF, P=0.055, but this did not reach significance (Fig. 1D). BDNF was also increased with Dukes staging [normal (2312±171) versus Dukes A 314±195, P=0.031; normal versus Dukes B and C (32761326), P=0.025]. Immunohistochemical staining showed only a small increase in BDNF protein in tumor tissues, compared with that in normal background colorectal tissues (Fig. 1E).

### mRNA expression of BDNF in human colon cancer cell lines

**Figure 2:**
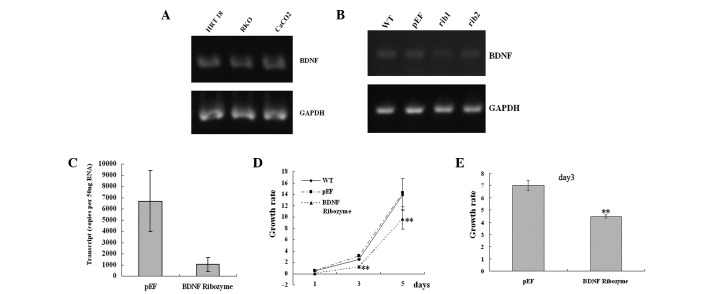
BDNF expression in human colon cancer cell lines and cell growth analysis. (A) Detection of BDNF mRNA transcription in Caco-2, HRT18 and RKO colon cancer cell lines using RT-PCR. GAPDH was used as the housekeeping control. (B) Knockdown of BDNF expression in Caco-2 cell lines. RT-PCR showed BDNF suppression at the mRNA level in Caco-2 cell lines. (C) Knockdown of BDNF expression in HRT18 cell lines. qPCR showed BDNF suppression at the mRNA level in HRT18 cell lines. (D) Knockdown of BDNF inhibited the Caco-2 cell growth compared with that of empty vector (pEF) control cells. (E) Knockdown of BDNF inhibited HRT18 cell growth compared with the control. ^**^Significantly different, P=0.0001 BDNF, brain-derived neurotrophic factor.

Human cancer cell lines Caco-2, HRT18 and RKO were examined for the presence of BDNF using RT-PCR (Fig. 2A). BDNF was strongly expressed in all three cell lines. Fetal kidney tissue was used as a positive control. The negative control had no DNA template (data not shown).

### BDNF knockdown and establishment of stable cell lines

The ribozymes targeting BDNF were cloned into pEF6/V5-his-Topo T/A vector. Caco-2 and HRT18 wild type cells were subjected to transfection using plasmids containing ribozymes targeting BDNF or an empty vector control, respectively, followed by the establishment of BDNF knockdown sub-lines and empty vector (pEF) control cells. The expression of BDNF at the mRNA level was reduced in BDNF knocked-down HRT18 and Caco-2 cells using RT-PCR and qPCR (Fig. 2B and C). We then characterized the effects of BDNF knockdown in these cells through a series of *in vitro* studies.

### Effects of BDNF knockdown on the growth of human colon cancer cells

In the *in vitro* growth assay, knockdown of BDNF in Caco-2 and HRT18 cells resulted in a reduction of cell growth rate (growth rate in BDNF knocked-down Caco-2 cells by day 3 Rib=1.24±0.15, compared with 3.15±0.12 in pEF, P=0.0000; Fig. 2D). Loss of BDNF in HRT18 cells resulted in reduction of cell growth rate (growth rate in BDNF knocked-down HRT18 cells by day 3 Rib=4.47±0.13, compared with 7.00±0.43 in pEF, P=0.0001; Fig. 2E). These data demonstrate that BDNF may increase colon cancer cell growth.

### Effects of BDNF knockdown on cell apoptosis

**Figure 3:**
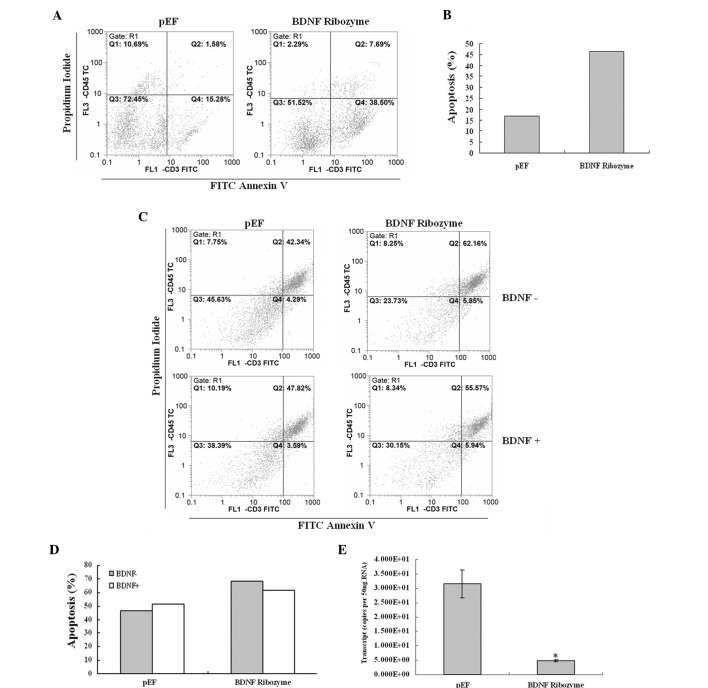
Effects of BDNF knockdown on cell apoptosis and the Bcl-2 pathway. (A) BDNF knockdown induces apoptosis in Caco-2 cell lines. Apoptosis was reduced compared with that of empty vector (pEF) control cells. (B) Statistical analysis of apoptosis. (C) BDNF knockdown induces apoptosis in HRT18 cell lines which was reversed using rescue experiments following exposure to BDNF (50 ng/ml) for 48 h. Apoptosis was reduced compared with that in pEF control cells. (D) Statistical analysis of apoptosis rescue using BDNF recombinant protein. (E) Effects of BDNF knockdown reduced the mRNA expression level of bcl-2 as assessed using RT-PCR. ^*^Significant difference of pEF vs, BDNF ribozyme, P<0.001 BDNF, brain-derived neurotrophic factor.

To investigate whether apoptosis is involved in the effect of BDNF knockdown in Caco-2 and HRT18 cells, we determined the proportion of apoptotic cells. As shown in Fig. 3A–D, there was an increase in cell population towards apoptosis in the BDNF knocked-down Caco-2 and HRT18 cells, which was 46.19% in the BDNF knocked-down Caco-2 cells, compared with 16.86% in the pEF control. There was also an increase of apoptosis in the BDNF knocked-down HRT18 cells, which was 68.01% in the BDNF knocked-down cells, compared with 46.63% in the pEF control. These results suggest that BDNF decreases apoptosis in these cells.

In addition, we confirmed whether or not this effect was specific to BDNF knockdown by rescue experiments. BDNF protein was added in the cell culture medium (50 ng/ml) and resulted in a negated effect of BDNF knocked-down HRT18 cells compared with the pEF controls (Fig. 3C and D).

### Effects of BDNF knockdown on cellular signal pathways

We screened the cells at the mRNA transcript level for bcl-2 in BDNF knocked-down HRT18 cells using qPCR. The results showed that the level of bcl-2 was reduced in BDNF knockdown HRT18 cells, indicating that BDNF stimulates the message for bcl-2. (Fig. 3E).

## Discussion

BDNF has been observed to be elevated in non-nervous system solid tumors including colorectal cancer. However, the impacts of BDNF transcription level on cellular biological function and the molecular pathways induced by BDNF are unknown (15). In our patient cohort, we found BDNF to have significantly elevated levels in tumors with poor prognosis and, to the best of our knowledge, this is the first study of its kind.

The expression levels of BDNF and TrkB mRNA have been demonstrated to be higher in human cancer cell lines than in normal tissues (16). Our results also show that the mRNA expression level of BDNF in human colon cancer is elevated. Therefore we utilized RNA knockdown experiments to study the effects of BDNF expression on cellular function and the possible molecular mechanisms. In the present study we obtained stable knockdowns of BDNF in human colon cancer cell lines using anti-BDNF ribozymes.

When BDNF was stably knocked down in Caco-2 and HRT18 cell lines, the growth decreased compared with that of cells transfected with the vector control, suggesting that reduced BDNF gene expression inhibited cellular proliferation. These results indicate that BDNF facilitates the proliferation of human colon cancer cells.

The apoptosis experiments demonstrated that in the Caco-2 and HRT18 cell lines in which BDNF was stably knocked down, apoptosis increased compared with that of the cells transfected with the vector control. In addition, we observed that when BDNF was added the apoptosis increased in HRT18 cells in which BDNF was stably knocked down. These results suggest that BDNF is a regulator of apoptosis in these cells. We conclude that BDNF is able to maintain colon cancer cell survival and proliferation.

The effect of BDNF on cellular biological functions is induced mainly by its receptor TrkB. When BDNF binds to TrkB the tyrosine kinase activity of the receptor is activated via phosphorylation of tyrosine residues in the cytoplasmic region of the receptor, which in turn induces cellular signaling (10,17).

The PI3K-AKT pathway is closely associated with cell survival and PI3K plays a key role in anti-apoptotic survival and proliferation (9,18,19). BDNF activates the AKT pathway in order to maintain cell survival (20). In this study, we also investigated the expression of downstream molecules associated with the AKT pathway. Bcl-2 as a member of the bcl-2 family, negatively regulates cell death and acts as an anti-apoptosis factor. Bcl-2 was downregulated in the BDNF knocked-down HRT18 cells compared with the level in the cells transfected with vector control. Accordingly, BDNF induces the increased transcription of bcl-2 to inhibit apoptosis and facilitate survival. BDNF downregulation eliminated protection from apoptosis, likely via the BDNF-Akt-Bcl2 anti-apoptotic signaling pathway (19).

In conclusion, our study shows that BDNF facilitates cell proliferation and inhibits cell apoptosis in human colon cancer cells. Reduced BDNF expression induces changes in downstream signaling molecules that are associated with cell survival and apoptosis. BDNF is therefore a potential therapeutic target in colon cancer and its effect in human colon cancer requires further investigation.

## Figures and Tables

**Table I:** Median values of BDNF expression in patient samples.

Clinical/pathological features	Sample (No.)	Median	Q1–Q3	P-value
Normal	68	0	0–7	
Tumor	88	25.5	2–438	<0.0001
Paired N	58	0	0–9.4	
Paired T	64	13	1–261	0
TNM				
Normal	68	0	0–7	
TNM1	9	163.4	39–1545	0.0001
TNM2	27	11	1–458	<0.0001
TNM3	25	7	1–175	0.0006
TNM4	6	244	1–939	0.0095
TNM2&3	52	10	1–246	0.0934
T1234				
Normal	68	0	0–7	
T-1	2	1491	NC	0.1435
T2	9	451	66–9953	<0.0001
T3	37	5	0–107	0.0001
T4	18	53	6–1058	<0.0001
T2	9	451	66–9953	
T3	37	5	0–107	0.0033
T2	9	451	66–9953	
T3&4	55	11	1–261	0.0086
Duke				
Normal	68	0	0–7	
Dukes A	7	74	20–451	0.0012
Dukes B	30	13	2–610	<0.0001
Dukes C	31	11	1–458	0.0001
Dukes BC	61	11	1–481	<0.0001
Alive	33	7	0–175	
Deceased	22	82	7–744	0.0554

BDNF, brain-derived neurotrophic factor. NC, not calculated.

**Table II:** Mean values of BDNF from patient samples.

Clinical/pathological features	Sample (No.)	Mean	SE Mean	P-value
Normal	68	212	171	
Tumor	88	2797	1003	0.013
Paired N	58	50	26	
Paired T	64	2224	887	0.017
Differentation				
Normal	68	212	171	
Diff 1	2	47.1	27	0.340
Diff 2	50	1918	785	0.039
Diff 3	14	7574	5023	0.170
Diff 2&3	64	3155	1265	0.024
T1234				
Normal	68	212	171	
T-1	2	1491	1491	0.550
T2	9	9594	7107	0.220
T3	37	2601	1307	0.078
T4	18	914	499	0.200
T2&3	46	3969	1740	0.037
T3&4	55	2049	896	0.049
T2&3&4	64	3110	1266	0.027
Normal	68	212	171	
No Invasion	48	1770	931	0.110
Invasive	24	4989	2822	0.100
Disease free	32	4757	2406	0.069
Incidence	23	1874	1021	0.120
Alive	33	3728	2233	0.130
Deceased	22	3219	1559	0.069

BDNF, brain-derived neurotrophic factor.
